# Lightweight Digital Certificate Management and Efficacious Symmetric Cryptographic Mechanism over Industrial Internet of Things

**DOI:** 10.3390/s21082810

**Published:** 2021-04-16

**Authors:** Adel A. Ahmed

**Affiliations:** Faculty of Computing and Information Technology, King Abdulaziz University, Rabigh, Jeddah 25729, Saudi Arabia; aaaabdullah1@kau.edu.sa; Tel.: +966-563884738

**Keywords:** digital certificate, ECDH, IIoT, symmetric cryptographic

## Abstract

The certificate authority, a trusted entity, issues digital certificates which contain identity credentials to help Industrial Internet of Things (IIoT) devices to represent their authenticity in a secure means. The crucial challenge of a digital certificate is to how design a secure certification authority management system that can counteract cyberattacks on the IIoT network. Moreover, current IIoT systems are not capable of implementing complex mathematical operations due to their constrained power capacity and processing capability. This paper proposes an effective, secure symmetric cryptographic mechanism (ESSC) based on the certificate authority management and Elliptic Curve Diffie Hellman (ECDH) to share a digital certificate among IIoT devices. The proposed certificate authority is used to securely exchange the shared secret key and to resolve the problem of spoofing attacks that may be used to impersonate the identity of the certificate authority. Also, ESSC uses the shared secret key to encrypt the sensitive data during transmission through the insecure communication channel. This research studies the adversary model for ESSC on IIoT and analyzes the cybersecurity of ESSC in the random oracle model. The findings that result from the experiments show that ESSC outperforms the baseline in terms of communication, computation, and storage costs. ESSC thus provides an adequate lightweight digital certificate management and cryptographic scheme which can help in the detection and prevention of several cyberattacks that can harm IIoT networks.

## 1. Introduction

The Industrial Internet of Things (IIoT) is a system/framework of smart devices that provide internet connection and communication capabilities to electronic devices, sensors, mechanical and digital machines, instruments, and any manufacturing objects used by industries. The IIoT devices have the capability to collect data and communicate with each other to enable intelligent industrial operations and achieve high productivity without requiring human intervention [1,2]. However, a non-negligible number of devices in IIoT networks are vulnerable to cybersecurity attacks, for example, device hijacking or spoofing, denial of service, man-in-the-middle, and data breaches. The effects of IIoT cyberattacks can cause catastrophic consequences for the investments of the business leaders who choose to implement IIoT. Hence, IIoT systems based on lightweight cryptography and efficacious digital signatures are essential to many models of cybersecurity protection. The recent IIoT cryptographic schemes can be classified into two types: symmetric (private key) and asymmetric (public key) cryptographic mechanisms. Symmetric cryptography uses a single (private) key to encrypt and decrypt a message. The strength of symmetric schemes truly depends on the distribution of the key between the IIoT devices [3,4,5,6,7]. In contrast, asymmetric cryptography uses two different (mathematically related) keys which are private and public keys. The private key is never distributed through the IIoT network. The public key can be announced through a secure channel to legitimate devices of the IIoT network. Unfortunately, the asymmetric or symmetric cryptography alone cannot be used to provide data integrity and sender authenticity. Therefore, a digital signature that is combined with symmetric/asymmetric cryptography can provide data confidentiality, sender authenticity, and data integrity. However, digital signatures have a crucial vulnerability in confirming the true identity of the sender. Digital signatures only prove that the private key of the sender which could be the imposter private key was used to encrypt the digital signature, but they do not absolutely prove the genuineness of the sender. Furthermore, the implementation of asymmetric algorithms on IIoT is more complex and utilizes more time and energy consumption compared to the symmetric algorithms.

One of the popular implementations of cryptography is digital certificates. In IIoT, a digital certificate is used to associate an object’s (a sensor, actuator, or user) identity to a public key using the digital signature of a trusted third party. The trusted third party has the capability to verify the owner’s identity and associate his public key with a digital certificate. When a remote IIoT user sends a message to an object, he does not ask it to retrieve his public key from IIoT gateway; instead, he attaches the digital certificate to that message. Upon the object receiving the message with the digital certificate, it can verify the digital signature of the trusted third party on the certificate. If the signature was signed by IIoT gateway, then it can be safely assumed that the public key contained in the digital certificate is actually from the legitimate IIoT user. Therefore, digital certificates make it possible for the object to verify user’s claim that the key belongs to him and prevent a spoofing attack that impersonates the public key of the owner [8,9].

In this research, a certificate authority center (CAC) at IIoT gateway serves as the trusted third-party agency that is responsible for issuing, distribution, status viewing and recovery of the digital certificates. The general duty of the CAC is to associate IIoT device identities to a public key and digitally sign the sensitive information using his private key. The owner of the digital certificate can be verified using the public key of CAC [10]. Furthermore, CAC can generate and publish certificate status information, maintain the security and availability, revoke public key certificates, and continuity of the certificate issuance signing functions. 

Regardless of the platform design of IIoT, the network model of IIoT is susceptible to numerous cyberattacks at all layers of TCP/IP model including application layer, network layer, and sensing layer. The cyberattacks exploit the vulnerability of IIoT system to harm, interrupt, gain unauthorized access to the sensitive information; or interrupt the production processes which may decrease the IIoT benefits. The IIoT cyberattacks might include physical attacks, device hijacking or spoofing, denial of service, man-in-the-middle, botnets, and data breaches. Unfortunately, IIoT platforms do not have security standard protocols that can defend against the aforementioned cyberattacks. Moreover, the standard TCP/IP cryptosystem imposes an essential computation cost because of the complex mathematical operations that must be executed in the encryption and decryption phases. Therefore, efficiently developing fast, compact, and secure cryptographic mechanisms for the IIoT is a demanding task. The developer of the cybersecurity mechanism on IIoT platform should design lightweight and efficacious mechanisms to prevent the disclosure of sensitive information to unauthorized attackers and to verify access to the IIoT services [11,12].

### 1.1. Problem Statement and Motivation

Asymmetric cryptography in conjunction with digital signatures has a crucial weakness in the confirmation of the true identity of the sender which means the private key of the sender that was used to encrypt the digital signature could be an impostor private key [13]. Furthermore, the implementation of asymmetric algorithms in IIoT is more complex and utilizes more time and energy. However, distributing and maintaining a secure single key among multiple users, who are often scattered geographically, poses significant challenges. This paper intends to investigate an effective, secure symmetric cryptographic mechanism based on digital certificate management and ECDH over Industrial Internet of Things networks. 

### 1.2. Summary of Contributions

This paper reports the following contributions:

- This research proposes an effective certificate authority management based on ECDH which is used to create a shorter digital certificate that is more suitable for resource-constrained devices. The proposed system can verify the true identity of the sender and provide protection against impersonation by binding a public key to its owner.
- This research proposes a symmetric cryptographic mechanism based on lightweight and effective mathematical operation on ECDH. It resolves the problem of exchanging secret key through the insecure communication channel and offers an efficient computation and communication costs, less execution (processing) overhead and storage.
- This research studies the adversary model for ESSC on IIoT and analyzes the cybersecurity of the proposed ESSC in the random oracle model which is considered a standard proven security method.

Finally, several simulation experiments have been conducted to evaluate the performance of the proposed ESSC in terms of communication, computation, and storage costs. The rest of this paper is structured as follows: Section 2 describes the related works on cryptographic and digital certificate algorithms over IIoT. The system design of the proposed ESSC algorithm is described in Section 3. Section 4 presents the threat model and cybersecurity analysis of ESSC mechanism. Section 5 explains the implementation of ESSC on IIoT and Section 6 presents the limitations of Implementation ESSC on IIoT. Finally, the conclusion and future work are explained in Section 7.

## 2. Related Work on Cryptographic and Digital Certificate Algorithms on IoT

Although many researchers have studied security algorithms in IIoT, a limited amount of research has benn focused on the development of effective, lightweight cybersecurity algorithms that target resource-constrained devices, especially for the sensors and actuators in IIoT networks. Thus, the overview of related works in this paper focuses on research studies that develop lightweight digital certificates, digital signatures and cryptographic algorithms for IIoT networks.

### 2.1. Lightweight Digital Certificates and Signatures in IoT systems

Transport Layer Security (TLS) and Datagram Transport Layer Security (DTLS) offer communications security for IoT that can prevent eavesdropping, tampering, and message forgery [14]. The relevant features of lightweight digital certificates for resource constrained IoT devices were first proposed by Forsby et al. [15], who developed a lightweight version of the X.509 (LX.509) certificate for IoT which provided compression and encoding schemes for the profiled certificate. An important feature is the compatibility with the X.509 standard which can be used in any existing PKI solution.

Elliptic curve cryptography (ECC) has been utilized in several cryptographic algorithms, including the elliptic curve digital signature algorithm (ECDSA [16]) which is a cryptographic public-key algorithm. Muhammad et al. [17] proposed a so-called shortened complex digital signature algorithm (SCDSA) for securing communication between smart devices in human-centered IoT applications. The research presented by Yasir et al. [18] proposed a lightweight security mechanism based on ECC and ElGamal for encryption and decryption over public-key (EEoP) infrastructures. Furthermore, Alizai et al. [19] developed a secure multi-factor authentication method which uses digital signatures and device capability to authenticate a device on IoT. Sciancalepore et al. [20] also developed a key management protocol (KMP) which combines implied certificates with ECDH exchange for verifying authentication and key generation.

### 2.2. Lightweight Cryptographic Algorithms on IoT

Elliptic Curve Integrated Encryption Scheme, or ECIES, is a mixed encryption system proposed by Victor Shoup in 2001. ECIES is combined with advanced standard encryption and called ECIES_AES, whereby a symmetric key from Elliptic Curve Cryptography (without the need for the Diffie-Hellman exchange) is created and used in encryption with 256-bit AES in ECB mode. Also, ECIES is combined with Rabbit and called ECIES_Ra). Rabbit is a standard stream cipher encryption protocol that has been designed for high performance software and described in RFC4503. NIST reports many lightweight authenticated encryptions with associated data (AEAD) ciphers which have been developed recently to deal with the needs of resource-constrained devices such as IoT systems [21]. AEAD provides data confidentiality, integrity and authentication. For instance, Seok et al. [22] designed secure D2D communication based on ECC and AEAD ciphers to cover resource-constrained IoT devices. Tokens have been used as the ECDSA with the associated data in the secure data communication step. The research presented by Muhammad et al. [23] proposed a lightweight encryption algorithm named Secure IoT (SIT) which is a mixture of Feistel and a uniform substitution-permutation network which requires a 64-bit key to encrypt the data. Also, Rajesh et al. [24] proposed a tiny symmetric encryption algorithm (NTSA) which adds a dynamic key confusion for each round of encryption for the transfer a text files through the IoT network. The combination of authentication and cryptography has been presented in Shah et al. [25] in which they proposed a combination of encryption algorithms, Diffie–Hellman, and a multifactor authentication system to share a secret key over the network. Shivraj et al. [26] proposed a lightweight one-time password (OTP) scheme based on Identity-Based Elliptic Curve Cryptography (IBE-ECC). However, IBE-ECC depends on a pre-shared key-based Diffie-Hellman exchange which is not enough to create secure encryption. Hammi et al. [27] proposed OTP that relies on Elliptic Curve Cryptography and Isogeny. Ayoub et al. [28] proposed a secure authentication and encryption based on improved ECC which is an asymmetric encryption scheme based on user credentials and biometric parameters. Also, Adeel et al. [29] developed a lightweight authentication algorithm based on elliptic ElGamal encryption. The authors combined the public key infrastructure (PKI) and ECC to generate a key pair and to exchange the secret key among IoT devices. Table 1 summarizes the most related research works and the limitations of each study.

The limitations of previous literature studies [14,15,16,17,18,19,20,21,22,23,24,25,26,27,28] can be divided into three types: Firstly, most of these research studies did not consider the hardware resource constraints and the appropriate architecture of IoT in the design of digital signature and cryptographic schemes is not considered. Secondly, the vulnerabilities of ECDH on IIoT were not covered and investigated as well. Finally, the divergence of IIoT devices’ abilities was not carefully taken into the design of the digital certificate and cryptographic mechanisms.

## 3. System Design of ESSC Algorithm

The proposed cybersecurity mechanism primarily consists of digital certificate management, and symmetric cryptographic algorithms which will ensure a high degree of cybersecurity protection against cyberattacks over IIoT.

### 3.1. Digital Certificate Management Algorithm 

The main purpose of digital certificate management is to securely exchange the shared secret key and to resolve the problem of spoofing attacks that are used to impersonate the identity of CAC. The proposed digital certificate management on IIoT consists of three functions which are management of issuance digital certificate, management of distribution digital certificate, and management of digital certificate recovery that will resolve the most critical certificate authority issues on IIoT networks. In order to design the three proposed functions, the following assumptions are made throughout this paper:

- The IIoT gateway (CAC) has a robust security mechanism which cannot be compromised by any attacker.
- The public key of the CAC and the domain parameters of the Elliptic Curve Diffie Hellman (ECDH) are embedded and uploaded to all IIoT devices during a programming session.
- The content of the digital certificate includes the certificate ID, public key of the digital certificate owner, ID of IIoT device, expiration time, and the digital signature of CAC for all fields of the certificate.

The following subsections describe the three algorithms and explain how these algorithms are used to secure the IIoT networks. All the notation used in this research is summarized in Table 2.

#### 3.1.1. Issuance and Distribution of Digital Certificate

**Figure 1:**
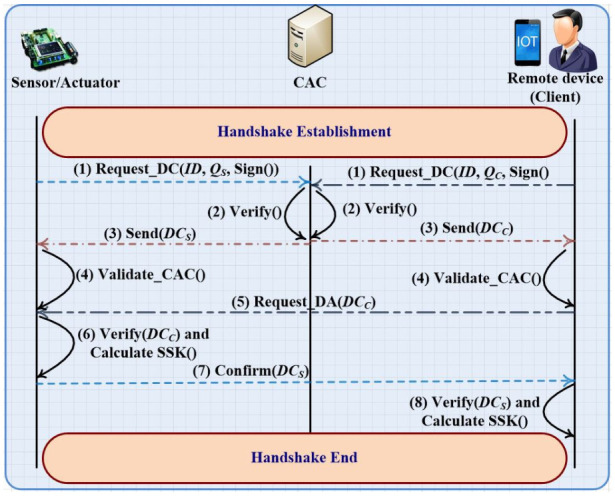
Issuance of digital certificate and handshake establishment algorithm.

The standard certificate authority mechanism is designed based on an asymmetric cryptography concept. This means the CAC should sign the digital certificate with his private key and the third party can verify any digital certificate using the public key of the CAC. However, the proposed certificate authority mechanism is designed based on Elliptic Curve Diffie Hellman (ECDH) which is used to create a shorter shared secret key that is more suitable for resource-constrained devices compared to other cryptographic algorithms (e.g., RSA). The elliptic curve is a set of points that is defined by the solution of the following equation:

(1) $$E=\;\left\{(x,y)|y^{2}=x^{3}+ax+b\right\}\cup \left\{O\right\},\;a,b\in K(\mathbb{Z}/p\mathbb{Z})\;\mathrm{Satisfy}\;(4a^{3}+27b^{2})\neq 0$$

where *K* is a finite field of integer numbers, modular prime *P*; *O* represents an extra point at infinity of the curve. Initially, the domain parameters *p*, *a*, *b*, *G*, *n*, and *h* represent the public information that should be agreed among CAC, sensor, actuator, remote IIoT user, etc. The parameter *p* specifies the prime of the base finite field of the curve (modulo *p*), *G* is the base point generator, *n* is the order of *G*, and *h* is the subgroup cofactor. In the proposed system, the CAC and the IIoT devices must have a key pair containing of a private key *d* (a randomly nominated integer number between 1, and *n −* 1) and a public key represented by a point *Q* (where *Q* = *d* × *G*, that is, the result of adding *G* to itself *d* times). Figure 1 illustrates the proposed time diagram of the issuance digital certificate and the handshake procedure that are used to create the shared secret key. The IIoT devices should request a digital certificate (RDC) from the CAC. The RDC contains information identifying the IIoT device (such as *ID*, *Q*) which must be signed using the IIoT’s private key. The CAC must verify the true identity of the sender using the digital signature included in the RDC and the verification of the public key as a valid curve point using three steps: 1. Check that *Q* is not equal to the identity element *O*, 2. Check that *Q_S/C_* is a point on the curve, and 3. Check that *n* × *Q_S/C_* = *O*. After the successful verification of the true identity of the sender, the CAC replies to each request with the appropriate digital certificate (*DC*). Upon receiving the *DC*, the IIoT device will use the stored value of *Q_C_* to verify the signature of the CAC that is appended in the received *DC*. Let us assume that the remote IIoT device wants to create a direct access (DA) to the sensor or actuator (usually the sending and receiving packets pass through the IIoT gateway); he will issue a handshake request that contains his *DC_C_* to the sensor or actuator. Upon receiving the handshake request, the sensor or actuator will verify the identity of the sender using the *DC_C_*. Furthermore, the sensor or actuator will calculate the shared secret key and will send it to the sender of the handshake request as (*X_K_*, *Y_K_*) = *d_S_* ×*Q_C_*. In addition, the sensor or actuator will send the confirmation message that includes his *DC_S_* to the sender of handshake request. Upon receiving the confirmation message, the sender of the request (remote IIoT device) will verify the identity of the sensor or actuator and he will calculate the shared secret key as (*X_K_*, *Y_K_*) = *d_C_* × *Q_S_*. Finally, the shared secret key will be *X_K_* which is equal in both parties, because *d_S_* × *Q_C_* = *d_S_* ×*d_C_* × *G* = *d_C_* × *d_S_* × *G* = *d_C_* ×  *Q_S_*. It is interesting to note that  is denoted to the scalar elliptic curve point multiplication by a scalar. Moreover, *Q*, *d*, and *X_K_* are ephemeral (dynamic) which means they are changed based on establishing a new session between the source and the destination. Indeed, the ephemeral shared secret key is recommended by RFC8442 to provide important security properties for ECDH such as perfect forward secrecy (PFS) and key-compromise impersonation resilience. The *X_K_* is used as a master key to encrypt and decrypt the plaintext during the transmission session.

#### 3.1.2. Recovery of Digital Certificate 

**Figure 2:**
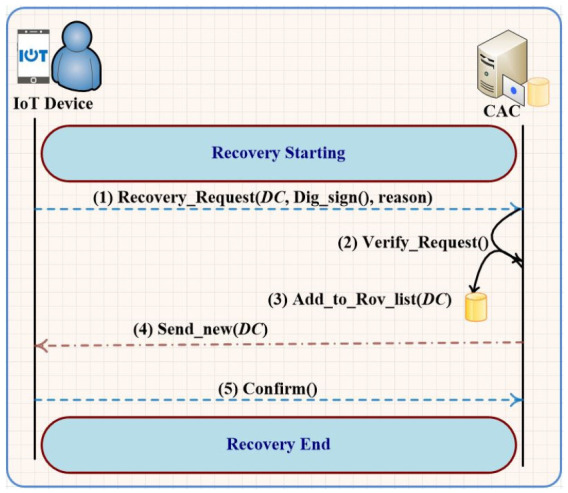
Digital certificate recovery algorithm.

The gateway has the capability to store the list of revoked digital certificates that might get lost, stolen, broken, compromised, expired, or revoked. Figure 2 depicts the procedure of the *DC* recovery which is started by sending a recovery request from an IIoT device (sensor, actuator, and IIoT remote user) to the CAC. The recovery request should include the revoked *DC*, the reason for the revocation, and the signature of the revocation request. The request verification in Figure 2 includes two verification procedures at the CAC: verification of the sender and validation of the revocation request. In first verification, the CAC will verify the correctness of the revocation request using the digital signature of the sender that must be included in the request. If the sender identity has been verified and the correctness of revoked *DC* have been validated, the CAC will add the revoked *DC* to the list of revocation *DC*s. Since the unique information of the revoked *DC* (e.g., *ID* of *DC* and the sender ID) and the expiration time of new *DC* have been stored at the CAC, the replaying/duplication of the certificate issuance request will be prevented. Finally, the CAC will create a new DC baseds on the received information and send it to the sender.

### 3.2. Lightweight Cryptographic Algorithm

The proposed secure symmetric cryptography uses ephemeral shared secret key that has been calculated from the previous step to be used in the encryption and decryption process which will prevent the disclosure of the sensitive information. The dynamic shared secret key is varied for each session which ensures forward secrecy protection. Furthermore, the proposed mechanism uses the scalar multiplication of the secure random number (*R*) and the *G* (the base point generator) to create a new point on the elliptic curve *E*(*X*_1_, *Y*_1_) which will be used to randomize the ciphertext for each message. 

The encryption algorithm is implemented at the source node using the following steps:

- Select a cryptographically secure random integer *R* between 1, and *n* − 1.
- Calculate *X_K_* as *K*(*X_K_*, *Y_K_*) = *d_S_* × *Q_D_*, where *d_S_* is the private key of the source and randomly nominated integer number between 1, and *n* − 1; *Q_D_* is the public key of the destination.
- *Calculate E*(*X*_1_, *Y*_1_) = *R*× G.
- *Compute T*= *X*_1_⊕*StrToInt*(Hash(*X_K_*) mod *n*), where Hash() is a cryptographic hash function, such as a CMA [30] or SHA-256 [31] and ⊕ is a bitwise XOR operation.
- Calculate *C*[0 … i] = *T* × *m*[0 … i] mod *n*, where *m*[0 … i] is the converting of the plaintext message (*M*[0 … i]) to the integer number using an agreed-upon reversible protocol known as a padding scheme. Each chunk (*M*) should include 24 bytes which is encrypted based on the elliptic curve (e.g., Secp192r1 that used in this research) [32].
- Send *C*[0 … i] and *X*_1_ to the destination.
- *X*_1_, and *X_K_* are randomly nominated in each session that is associated between the source and destination.

The decryption algorithm is implemented at the destination node using the following steps:

- Verify the public key of the sender (*Q_S_*) based on three steps of curve point inspection: 1. Check that *Q_S_* is not equal to the identity element *O*, 2. Check that *Q_S_* lies on the curve, and 3. Check that *n Q_S_* = *O*.
- Calculate *X_K_* as *K*(*X_K_*, *Y_K_*) = *d_D_* × *Q_S_*, where *d_D_* is the private key of the destination and it is randomly nominated integer number between 1, and *n* − 1.
- Compute *T = X1* ⊕ *StrToInt*(Hash(*X_K_*) mod *n*), where Hash() is the same function used in the encryption process.
- Calculate *m*[0 … i] = *C*[0 … i] × *T*^−1^ mod *n*, where *T*^−1^ mod *n* can be solved using a modular multiplicative inverse.
- Convert *m*[0 … i] back to the plaintext (*M*[0 … i]) and remove the padding bytes from last chunk *M*[i].

The pseudocode of ESSC algorithm is introduced in Algorithm 1. The destination will follow the same procedure as a source if it sends back a reply message. In that case, the source will act as the destination and the destination will act as the source. Algorithm 1 presents the pseudo code of the proposed algorithm. In this algorithm, the source and the destination nodes must use the proposed digital certificate to exchange the public key between the parties of communication. After that, the public key will be used to create the shared secret key. Each encrypted message is created based on a secure random number and a shared secret key; therefore, the ciphertext is different for a similar message which satisfies the ciphertext indistinguishability (IND-CPA) and it prevents the replay attacks.
**Algorithm 1: ESSC Algorithm at Source (S) Node**
Input: *DC*, the domain parameters *p*, *a*, *b*, *G*, *n*, *h;//DC*: Digital Certificate 
Output: *SSK*, *C;//SSK: Shared Secret Key*, *C*: Ciphertext
Start Algorithm (ESSC) 1| While (new session starts) do 2|   Pick private key (*d_S_*);//1 ≤ *d_S_* ≤ *n*3|   *Q_S_ = d_S_*
×
*G*);/*/Q_S_:* the public key of the source node 4|   Request_DC(*ID*, *Q_S_*, *Sing()*);//*Request a DC from CAC*5|   *CAC_Verify (RDC)*;//*CAC verifies the request and the identity of the sender; RDC: Request Digital Certificate.*6|   if (*DC_S_* is received);//*Receive DC_S_ from CAC*7|        Verify (*CAC*, *DC_S_*);//*Verify the DC_S_ and the CAC*8|   End9|   if (*DA* is received)//*Receive DA request from IIoT device*10|      Verify (*CAC*, *DC_C_*); //*Verify the DC_C_ sent from IIoT device*11|      Obtain (*Qc*);//Get the public key of IIoT device from *DC_C_*12|      SSK (*X_K_*,*Y_K_*) = *d_S_*
×
*Q_C_*; //calculate shared secret key (*X_K_*)13|   End14|   Pick Random Number (*R*);//1 ≤ *R* ≤ *n*15|     *E* (*X*_1_,*Y*_1_) = *R*
×
*G*; //calculate the curve point (*E*)16|     *T*
*= X*_1_
⊕ (*StrToInt*(Hash(*X_K_*)) mod *n*); 17|   if (Count(*M*) > 24)//count number of bytes in *M*, 24 (192 bits) which related to elliptic curve (Secp192r1) used in this paper 18|     *M*[0…i] = Split(*M*,*24);Pad(M(i)*,*24);//*Split *M* to number of chunks each 24 bytes, last chunk will be padded to be 24 bytes 19|     *m*[0…i] = StrToInt(*M*[0 … i]);*//*convert the plaintext to number; *m*[i] is the last part of converting of *M*[i] with padding 20|     *C*[0…i] = (*m*[0 … i] ×
*T*) mod *n*;//*C*: the ciphertext21|      Send (*C*[0 … i], *X*_1_);/*/Send* the ciphertext and *X*_1_22| End;//While loop 23End;//Algorithm 
ESSC Algorithm at destination (D) node
Input: *DC*, *C*[0 … i], *X*_1_, the domain parameters *p*, *a*, *b*, *G*, *n*, *h*;//*DC*: Digital Certificate, *C*: Ciphertext 
Output: *M*[0 … i], *SSK*;//*SSK: Shared Secret Key*,
Start Algorithm (ESSC) 1| While (new session starts) do 2|    Pick private key (*d_C_*);//1 ≤ *d_C_* ≤ *n*3|    *Q_C_ = d_C_*×*G*;/*/Q_S_:* the public key of the source node 4|    Request_DC(*ID*, *Q_C_*);//*Request digital certificate from CAC*5|   *CAC_Verify (RDC)*;//*CAC verifies Request Digital Certificate*6|    if (*DC_C_* is received*_)_*;//*Receive DC_C_ from CAC*7|      Verify (*CAC*, *DC_C_*);//*Verify the DC_C_ and the CAC*8|      End9|      Send_DA (S)//Send DA request to the Source (S)10|      if (DA Confirm is received)//Receive DA Confirm from S11|      Verify (CAC, DC_S_);//Verify the DC_s_ sent from IIoT device 12|      Obtain (*Q_S_*);//Get the public key of the *S* from *DC_S_*13|      SSK (*X_K_*,*Y_K_*) = *d_C_*
×
*Q_S_*; //calculate shared secret key (*X_K_*)14|        End15|       if (*C*[0 … i] and *X*_1_ are received)//*D receives C*[0 … i] *and X*_1_16|      Check (*Q_S_*);//check public key of the *S* is a curve point 17|      *T* = *X*_1_
⊕ (*StrToInt*(Hash(*X_K_*)) mod *n*); 18|       *m*[0 … i] = (*C*[0 … i] ×
*T^−1^*) mod *n*;//*m*: the ciphertext19|  *M*[0 … i] = Convert_IntToStr(*m*[0 … i]); *Remove_pad(M(i),24);*20|        End21| End;//While loop22End;//Algorithm 

## 4. Adversary Model and Cybersecurity Analysis

In order to evaluate the security assurance of ESSC, an adversary model is defined based on random oracle model to simulate the cyberattacks on ESSC and exploit the vulnerabilities of IIoT.

### 4.1. Adversary Model for ESSC on IIoT

The adversary model consists of possible attacks that can be implemented using external or internal adversary on ESSC based IIoT. In the cryptanalysis, the adversary is assumed to have the capability to breach ESSC cryptographic security system and gain access to the contents of encrypted messages, even if the shared secret key is unknown [33,34,35,36,37,38,39]. This research studies the following attacks’ model for cryptanalysis in the random oracle model (ROM):

- *Chosen-plaintext attack (CPA).* It presumes that the adversary can obtain the ciphertexts for arbitrary plaintexts. In the adaptive CPA (CPA2), the adversary can make his choice of the inputs to the encryption function of ESSC based on the previously chosen plaintext queries and their corresponding ciphertexts [38]. Let an adversary ***A*** has access to an oracle with any pair of equal-length messages (***m*_1_**, ***m*_2_**) as input. The oracle will return a ciphertext as output.

**Definition** **1.** *Let ESSC = (K, E, D) be a symmetric encryption scheme, and **A** be an adversary that has access to the oracle. The IND-CPA advantage of A is defined as:*

(2) $$Adv_{{}_{\mathrm{ESSC}}}^{\mathrm{in-cpa}}\left(A\right)=P_{r}[k\leftarrow K;C\leftarrow E_{k}\left(m_{1}\right):A(C)=1]-P_{r}[k\leftarrow K;C\leftarrow E_{k}\left(m_{2}\right):A(C)=1]$$

*If the advantage is small, it means that is not doing well and ESSC is secure. In contrast, if the advantage is large, it means that is doing well and ESSC is not secure.*

- *Chosen-ciphertext attack (CCA).* It presumes that the adversary can obtain the decryption of any ciphertext(s) of its choice. In the adaptive CCA (CCA2), the adversary can make his choice of the inputs to the decryption function of ESSC based on the previously chosen ciphertexts queries [39].

**Definition** **2.** *Let ESSC = (K, E, D) be a symmetric encryption scheme, and **A** be an adversary that has access to the E and D oracle. The IND-CCA advantage of A is defined as:*

(3) $$Adv_{{}_{\mathrm{ESSC}}}^{\mathrm{in-cca}}\left(A\right)=P_{r}[k\leftarrow K;C\leftarrow E_{k}\left(m_{b}\right);b\leftarrow \left\{0,1\right\};b^{\prime }\leftarrow A\left(E_{k}\left(.\right),D_{k}\left(.\right)\right):b^{\prime }=b]$$

*The adversary’s access to the decryption oracle is unlimited except for the restriction that the adversary should not request a query to the decryption oracle because **C** was previously returned by the encryption oracle. ESSC is secure against IND-CCP if a “reasonable” adversary cannot obtain “significant” advantage in distinguishing the cases b = 0 and b = 1 given access to the oracles, where reasonably reflects its resource usage.*

- *Related-keyattacks.* The adversary knows or chooses a mathematical relation between several keys and is given access to encryption functions (*E*) of ESSC with such related keys. The goal of the attacker is to find the actual secret key(s) (*X_K_*). Let us define Perm(*K*, ${\mathbb{Z}}_{n}$) as the set of all block-ciphers with domain ${\mathbb{Z}}_{n}$ and key-space *K*. Moreover, let Φ be a set of functions that map *k* to *K*. We call Φ the set of allowed related-key-deriving (*RKD*) functions. Also, let us define the related-key oracle *E_rk(·,k)_*(.) on the *E* of ESSC as an oracle that takes two arguments, *ϕ* ∈ Φ and an element *M* ∈ ${\mathbb{Z}}_{n}$, and it returns *E_ϕ(k)_*(*M*). The pseudorandom permutation with respect to related-key attacks (*PRP-RKA*) can be defined as follows:

**Definition** **3.** *Let E: K × D→D be a family of functions and let Φ be a set of allowed RKD functions over K. Let adversary A has access to a related-key oracle, and restricted to queries of the form (ϕ, M) in which M ∈ ${\mathbb{Z}}_{n}$. Then the PRP-RKA advantage of A in a Φ-restricted related-key attack (RKA) on E is defined as:*

(4) $$Adv_{{}_{\Phi ,E}}^{\mathrm{prp-rka}}\left(A\right)=P_{r}[k\leftarrow K:A\left(E\left(\phi \left(k\right),M\right)\right)=1]-P_{r}[k\leftarrow K;G\leftarrow Perm\left(K,D\right):A\left(G\left(\phi \left(k\right),M\right)\right)=1]$$

The adversary model allows A to choose a function ϕ which transforms the target key K into the key ϕ(K), and then to obtain the value of the block cipher, on an input of A’s choice, under this transformed key. If the advantage is small, it means that ESSC is secure against Φ-restricted related-key attack. In contrast, if the advantage is large, it means that A is doing well and ESSC is not secure against Φ-restricted related-key attack.

### 4.2. ESSC Cybersecurity Analysis

ESSC is designed based on the digital certificate to share the ephemeral shared secret keys that are different for each session, and can provide important security properties such as PFS and key-compromise impersonation resilience. Indeed, the ephemeral shared secret key for ECDH is recommended by RFC8442. The cryptographically secure random that was multiplied with the base point in the elliptic curve is used to create random ciphertext indistinguishability (IND-CPA) and it prevents the replay attacks. This section explains the cybersecurity analysis for ESSC in ROM. The proven security of ESSC under ROM investigates the previous attacks on the proposed shared secret key-derivation, cryptography, and digital certificate algorithms.

#### 4.2.1. Proven Security for ESSC in ROM

Let us assume that the sender and receiver shared a secret random key such as $X_{K}\in {\left\{0,1\right\}}^{L}$ (where *L* = $\left|X_{K}\right|\;=\;\left|n\right|\;=\;\left|p\right|$; is the length of each domain parameter in Secp192r1 which is equal to 192 bits) and the base point as $G\left(x,y\right)\;\in E_{a,b}\left({\mathbb{Z}}_{n}\right)$. To encrypt message $M\in {\left\{0,1\right\}}^{L}$, the sender picks a random $R\in {\left\{0,1\right\}}^{L}$, computes $E\left(X_{1},Y_{1}\right)\;=R\times G\left(X,Y\right)$ and $T\left(X_{1},X_{K}\right)\;=X_{1}⊕H\left(X_{K}\right)\;mod\;n$, computes C=T×M modn, and sends $\left(X_{1},C\right)$ to the receiver. To decrypt the ciphertext $\left(X_{1},C\right)$, the receiver computes $T\left(X_{1},X_{K}\right)\;=X_{1}⊕H\left(X_{K}\right)\;mod\;n$ and $M=T^{-1}\times C\;mod\;n$. In order to prove the security of ESSC cryptography, the ROM is used to instantiate the hash function as $H\left(.\right):{\left\{0,1\right\}}^{*}\to {\left\{0,1\right\}}^{L}$.

**Theorem** **1.** *If T is a (t, ϵ)-pseudorandom function (PRF), then the ESSC cryptographic is secure in the sense of indistinguishability against CPA (secure against IND-CPA).*

**Proof.**  This theorem will be proven through the contradiction methodology in which we assume the encryption (*E*) of ESSC is not secure. This means there exists some algorithm probabilistic polynomial time (*PPT*) *A* that breaks it. We will show how to use such algorithm to construct a *PPT* distinguisher *B* which can distinguish the output of *T* from a random string with non-negligible probability. This will contradict the fact that *T* is *PRF*; hence, our original assumption is false and the encryption (*E*) of ESSC must be secure. Let us assume we have an adversary *A* attacking the encryption (*E*) of ESSC in the sense of IND-CPA and we have messages *M*_0_, *M*_1_ for which:

(5) $$\left|\begin{array}{l} P_{r}[H(X_{K})\leftarrow {\mathbb{Z}}_{{}_{n}}^{*};T\leftarrow X1⊕H(X_{K});C\leftarrow M_{0}\times T:A(C)=0]\\ -P_{r}[H(X_{K})\leftarrow {\mathbb{Z}}_{{}_{n}}^{*};T\leftarrow X1⊕H(X_{K});C\leftarrow M_{1}\times T:A(C)=0] \end{array}\right|\;=\gamma (L)$$

where γ(n) is not negligible. We construct an algorithm *B* which tries to distinguish *T* from the random function. The adversary *B* is given oracle access to *T* which is either a completely random function or *PRF*. *B* runs as follows: (1) Choose random $b\in \;\left\{0,1\right\}$, (2) *B* sets $C=T\times M_{b}\mathrm{mod}n$, (3) Run *A(C)* to get $b^{\prime }$ which represents *A*’s guess as to what message was encrypted. If b= $b^{\prime }$ (*A* guessed correctly) then *B* guesses pseudorandom which denoted by *B* outputs “1”. In contrast, If b≠ $b^{\prime }$ (*A* did not guess correctly) then *B* guesses random which denoted by *B* outputs “0”. The algorithm *B* distinguishes output of *T* as:

(6) $$\left|P_{r}[H(X_{K})\leftarrow {\mathbb{Z}}_{{}_{n}}^{*};y\leftarrow T(H(X_{K})):B(y)=1]-P_{r}[y\leftarrow {\mathbb{Z}}_{{}_{n}}^{*}:B(y)=1]\right|$$

Let us look at each of these terms individually as: *P*_1_ ≝ $P_{r}[H(X_{K})\leftarrow {\mathbb{Z}}_{{}_{n}}^{*};y\leftarrow T(H(X_{K})):B(y)=1]$ and *P*_2_ ≝ $P_{r}[y\leftarrow {\mathbb{Z}}_{{}_{n}}^{*}:B(y)=1]$. In step 3, the algorithmdid the following:

(7) $$P_{1}=P_{r}[H(X_{K})\leftarrow {\mathbb{Z}}_{{}_{n}}^{*};y\leftarrow T(H(X_{K})):b\in \{0,1\};b^{\prime }\leftarrow A(T\times M_{b}):b^{\prime }=b]$$

Conditioning on the value of b gives:

$$P_{1}=P_{r}[H(X_{K})\leftarrow {\mathbb{Z}}_{{}_{n}}^{*};y\leftarrow T(H(X_{K})):A(T\times M_{0})=0]\times P_{r}[b=0]+P_{r}[H(X_{K})\leftarrow {\mathbb{Z}}_{{}_{n}}^{*};y\leftarrow T(H(X_{K})):A(T\times M_{1})=0]\times P_{r}[b=1]$$

Using the fact: Pr[b=0]=Pr[b=1]=12, and

$$P_{r}[H(X_{K})\leftarrow {\mathbb{Z}}_{{}_{n}}^{*};y\leftarrow T(H(X_{K})):A(T\times M_{1})=1]=1-P_{r}[H(X_{K})\leftarrow {\mathbb{Z}}_{{}_{n}}^{*};y\leftarrow T(H(X_{K})):A(T\times M_{1})=0]$$

gives:

(8) $$\begin{array}{ll} P_{1} & =\frac{1}{2}+\left[\frac{1}{2}\times \left(P_{r}[H(X_{K})\leftarrow {\mathbb{Z}}_{{}_{n}}^{*};y\leftarrow T(H(X_{K})):A(T\times M_{0})=0]-P_{r}[H(X_{K})\leftarrow {\mathbb{Z}}_{{}_{n}}^{*};y\leftarrow T(H(X_{K})):A(T\times M_{1})=0]\right)\right]\\ & =\frac{1}{2}+\left(\frac{1}{2}\times \gamma (L)\right) \end{array}$$

$P_{2}$ is calculated as:

(9) $$P_{2}=P_{r}[y\leftarrow {\mathbb{Z}}_{{}_{n}}^{*}:b\in \{0,1\};b^{\prime }\leftarrow A(T\times M_{b}):b^{\prime }=b]$$

As before, we eventually get:

(10) $$P_{2}=\frac{1}{2}+{\left[\frac{1}{2}\times \left(P_{r}[y\leftarrow {\mathbb{Z}}_{{}_{n}}^{*}:A(T\times M_{0})=0]-P_{r}[y\leftarrow {\mathbb{Z}}_{{}_{n}}^{*}:A(T\times M_{1})=0]\right)\right]}_{\;}$$

Since *y* is completely random and $T\left(X_{1},X_{K}\right)=X_{1}⊕H\left(X_{K}\right)\;mod\;n$, the success probability of *A* when attacking the one-time pad is 0. Thus, $P_{2}$ is 1/2. Putting everything together gives:

(11) $$\left|P_{r}[H(X_{K})\leftarrow {\mathbb{Z}}_{{}_{n}}^{*};y\leftarrow T(H(X_{K})):B(y)=1]-P_{r}[y\leftarrow {\mathbb{Z}}_{{}_{n}}^{*}:B(y)=1]\right|=\left|P_{1}-P_{2}\right|=\left|\frac{1}{2}\pm \frac{\gamma (L)}{2}-\frac{1}{2}\right|=\frac{\gamma (L)}{2}$$

Since γ(L) was not negligible, $\frac{\gamma (L)}{2}$ is not negligible. This means that *A* had non-negligible advantage in breaking the encryption of ESSC and therefore *B* had non-negligible advantage in breaking the *PRF* (i.e., distinguishing output of from random). However, this contradicts the fact that *T is a (t,* *ϵ)-PRF* and our assumption must be wrong, and no such *A* can exist; hence, the encryption of ESSC is secure. ☐

**Theorem** **2.** *Let E: K × {0, 1}^L^→ {0, 1}^L^ be a block cipher and let ESSC = (K, E, D) be a symmetric encryption scheme, then for all PPT adversaries, the IND-CCA advantage of A is negligible in the ROM.*

**Proof.**  Let us assume there exists some algorithm *PPT A* that breaks ESSC in the sense of IND-CCA for which $Adv_{{}_{\mathrm{ESSC}}}^{\mathrm{in-cca}}\left(A\right)=1$. The encryption oracle $E_{K}$ (*m_b_*) takes input a pair of messages, and returns an encryption of either (*m*_0_, *m*_1_) message in the pair, depending on the value of *b*. The goal of *A* is to determine the value of *b* and it works as follows:

  ***A***(***E***(*m_b_*), ***D*** (·)) {

  $\;m_{0}\leftarrow 0^{L};m_{1}\leftarrow 1^{L};(X_{1},C)\leftarrow E_{K}\left(\left(m_{0},m_{1}\right),b\right);\;C^{\prime }\leftarrow C\;⊕\;1^{L};\;M\leftarrow D_{K}(X1,C^{\prime });$

  *If M = m*_0_ than return 1 else return 0}

The encryption oracle is queried with the pair of distinct messages (*m*_0_, *m*_1_), each one block long and the a ciphertext (*X*_1_,C) is returned. The adversary flips the bits of *C* to get $C^{\prime }$ and then feeds the ciphertext (*X*_1_,$C^{\prime }$) to the decryption oracle to obtain the message *M*. Finally, the adversary can flip the bits of *M* (i.e., the flip bits should be in same position that are flipped in *C*) to obtain the original message (plaintext). It is interesting to note that (*X*_1_,$C^{\prime }$) = (*X*_1_,C), so the decryption query is legitimate. The advantage of *A* in attacking ESSC with IND-CCA is defined as:

(12) $$Adv_{{}_{\mathrm{ESSC}}}^{\mathrm{in-cca}}\left(A\right)=P_{r}[{\mathrm{Exp}}_{ESSC}^{ind-ccp-1}(A)]-P_{r}[{\mathrm{Exp}}_{ESSC}^{ind-ccp-0}(A)]$$

Let us look at each of these terms individually, we start with ${\mathrm{Exp}}_{ESSC}^{ind-ccp-1}(A)$ (b = 1) as: $C=E_{K}\left(m_{1},b\right)=T\times m_{1}\mathrm{mod}n$. If we flip bit *i* of *C*, resulting in a new ciphertext $C^{\prime }$ and the decryption oracle with $C^{\prime }$ is queried as:

(13) $$M=D_{K}\left(X_{1},C^{\prime }\right)=T^{-1}\times \left[\left(T\times m_{1}mod\;n\right)⊕1^{L}\right]mod\;n=T^{-1}\times \left[\left(T\times 1^{L}mod\;n\right)⊕1^{L}\right]mod\;n$$

The modular multiplication does not distribute over XOR (e.g., $6\times \left(3⊕7\right)\neq \left(6\times 3\right)⊕\left(6\times 7\right)$) and it also does not associate over XOR (e.g., $6\times \left(3⊕7\right)\neq \left(6\times 3\right)⊕7$). Hence, *M*≠ *m*_0_ and the returned value is 0 which means *A* did not guess correctly and $P_{r}[{\mathrm{Exp}}_{ESSC}^{ind-ccp-1}(A)]$ is 0. Eventually, the second term can be proven as before in which $P_{r}[{\mathrm{Exp}}_{ESSC}^{ind-ccp-0}(A)$ is 0. Putting everything together gives $Adv_{{}_{ESSC}}^{in-cca}\left(A\right)$ is 0. Thus, The advantage of *A* in attacking ESSC with IND-CCA is negligible. ☐

**Theorem** **3.** *Let E: {0, 1}^L^× {0, 1}^L^→ {0, 1}^L^ be a block cipher and let ESSC defined as $\tilde{E}$ (K, X_1_,M) = E(KX_1_, M). Then given ESSC-PRP adversary A against $\tilde{E}$ we can construct $\Phi_{k}^{⊕}\left(\phi \left(K\right)\;=K⊕k\right)$-restricted PRP-RKA adversary B against E such that:*

(14) $$Adv_{{}_{\tilde{E}}}^{\mathrm{essc-prp}}\left(A\right)\leq Adv_{{}_{\Phi_{k}^{⊕},E}}^{\mathrm{prp-rka}}\left(B\right)$$

**Proof.**  Let *B* be an adversary that runs *A* and, when *A* makes an oracle query *M*, *B* makes oracle query (*ϕ*, *M*) and returns the response to *A* as follows:

  Adversary $B^{F_{RK\left(.,K\right)}\left(.\right)}$ {

   Run *A*, responding to *A*’s request (*X*_1_*, M*) as follows:

    Return $F_{RK\left(.,K\right)}\left(M\right)\{K^{\prime }\leftarrow K⊕X_{1}$

       *C* $\leftarrow E_{K^{\prime }}(M);$ (*M*); Return *C*;} to *A*;

   Until *A* halts returning a bit *b*; Return *b*;}

If *A* queries its oracle with *X*_1_ (*q* times), then *B* runs at the same time as *A* and queries its oracle with key transformation *ϕ(K)* (*q* times). We measure its success in determining whether its oracle queries are being answered via the block cipher $\tilde{E}$ or via a random block cipher. The following equality holds because *K*⊕ *X*_1_ is a permutation on *K* and *B* computes $\tilde{E}$ exactly:

(15) $$\;P_{r}[k\leftarrow K:A\left(\tilde{E}\left(k,X_{1},M\right)\right)=1]=P_{r}[k\leftarrow K:B\left(E\left(\left(k⊕X_{1}\right),M\right)\right)=1]$$

Furthermore, because for each *X*_1_ in *A*’s queries of *G*, *B* replies to *A* using an independently selected random permutation on {0, 1}^n^. This is mainly due to the fact that $\mathrm{InSe}c_{{}_{\Phi_{k}^{⊕}}}^{cr}\left(\left|\Phi_{k}^{⊕}\right|\right)$ = 0. Consequently, the following equality holds as well:

(16) $$P_{r}[G\leftarrow Perm\left(l,n\right):A\left(G\left(k,X_{1},M\right)\right)=1]=P_{r}[k\leftarrow K;G\leftarrow Perm\left(l,n\right):B_{A}\left(G\left(k⊕X_{1},M\right)\right)=1]$$

From Equations (15) and (16), Theorem 3 is proven. ☐

#### 4.2.2. Proven Security for Proposed Digital Certificate in ROM

Let us assume that RDC includes *ID*, *Q*, *Sing*(*d, Ms*) where $ID\in {\left\{0,1\right\}}^{*}$, $Q\in E_{a,b}\left({\mathbb{Z}}_{n}\right),\;d\in {\left\{0,1\right\}}^{L}$ and *Ms* is the *DC* data that should be signed. The output of digital signature function is $\sigma \in {\left\{0,1\right\}}^{*}$. Moreover, let us assume that the output of verifying function *VerfySign*(Q,*Ms*,σ) is 1 (valid) or 0 (invalid). Let $H\left(Ms\right):{\left\{0,1\right\}}^{*}\to D_{Q}$ (Domain of *Q*) be a hash function modeled as random oracle. To sign *DC*, the output σ = *Sign(d, H(Ms))*. Also, to verify signature σ on DC, we check that *Sign*^−1^(Q, σ) $\overset{?}{=}$ *H(Ms)*.

**Theorem** **4.** *If Sign function is a (t, ϵ)-secure, then the ESSC digital certificate is (t, q ϵ)-secure (unforgeable against adaptive CPA), where q represents the number of queries an adversary makes to the random oracle H.*

**Proof.**  This theorem will be proven through contradiction methodology same as Theorem 1. Assume there is some adversary *A* which outputs a forgery for the above construction with probability *δ*. We use this adversary to construct an algorithm *B* that inverts *Sign(d*, *H(M_S_))*. *B* is given *Q* and a random element *y*, and tries to compute *x* such that *Sign^−1^*(*Q, x*) = *y*. *B*(*Q*, *y*) proceeds as follows:

- Pick a random index i^*^ ∈ {1,…, q};
- Run A(Q) and Answer the ith query of A to oracle *H* as follows (let m_i_ denote ith query): if i = i^*^, return *y*; otherwise, pick random r_i_ ← *D_Q_*, compute ans_i_ = $Sign^{-1}\left(Q,r\right)$, and return ans_i_;
- When A requests a signature on message m: find i such that m = m_i_; if i = i^*^, abort; otherwise, return r_i_ as the signature.
- When A outputs its forgery (m, σ) If m = m_i*_ then output σ; otherwise, abort.

*B* sets things up so that it is able to answer all signature queries of *A* unless a signature on m_i*_ is requested. Note that the response of *B* to all other signature queries is indeed a correct signature. Since the output (*m*, *σ*) of *A* can only be a forgery if *A* never requested a signature on *m*, and since *m* = *m_j_* for some *j* (by assumption), it must be the case that there is at least one index *j* for which *A* never requests a signature on *m*_j_. Since i^*^ is chosen at random, with probability at least 1/*q* we have *j* = i^*^. When *j* = i^*^ (and *A* outputs a valid forgery) then *σ* is indeed an inverse of *y* and *B* succeeds in inverting the Sign function. However, this contradicts the fact that *Sign* function *is a (t,* *ϵ)-*secure and our assumption must be wrong, and no such *A* can exist; hence, the digital certificate is secure as well. To summarize the above discussion, the probability that *B* correctly outputs an inverse is at least 1/*q* times the probability that *A* outputs a forgery; hence, *B* outputs the desired inverse with probability at least *δ/q*. Since the *Sign* function is assumed to be *a* (*t,* *ϵ*) *-*secure, we must have *δ ≤ qϵ*.

#### 4.2.3. Countermeasure against Replay and Man-In-The-Middle Attacks

ESSC mechanism defends against the man-in-the-middle and replay attacks using the proposed digital certificate and secure random number in the cryptographic mechanisms. Moreover, ESSC will discard the replay attack message from the malicious nodes due to the following reasons:

- The authentication in the digital certificate should be inspected before accepting any data message from a man-in-the-middle. For more explanation, Section 5.1 discusses how the IIoT device prevents the replay message from the intruder.
- Each encrypted message is created based on a secure random number which satisfies the ciphertext indistinguishability (IND-CPA) property that will prevent the replay attacks.
- The ephemeral shared secret key is calculated and only known by the source and destination.

#### 4.2.4. Countermeasure against Brute Force Attacks

The proposed ESSC mechanism can defend against this attack using the PFS that can be obtained using the hash function of the ephemeral shared secret key. The shared secret key must change every communication session between parties in IIoT system. Furthermore, the encryption process relies on secure random key which complicates the brute force attack. The PFS prevents cracking the key-agreement protocol in ESSC which might be applied by the attacker with likely quite computationally intensive.

#### 4.2.5. Countermeasure against Stolen-Verifier and IIoT Device Capture Attacks

The ESSC algorithm preserves against those attacks using the embedded hash function and the public key of the CAC that used to calculate digital certificate and ciphertext in the proposed ESSC. The hash function and the CAC public key are parts of source code which is flashed into the IIoT device using the machine level language code during programming session. Thus, identified the shared secret key without knowing the hash function will prevent the intruder to gain access to the IIoT network.

## 5. Implementation and Evolution of ESSC on IIoT

**Figure 3:**
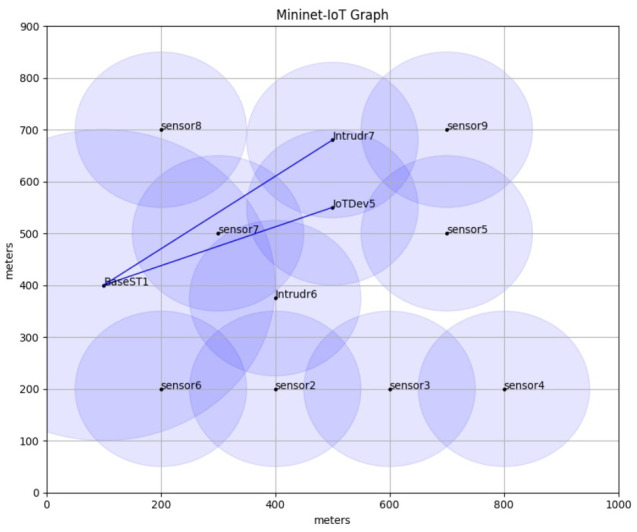
IoT mesh topology and ESSC implementation.

The hardware of a IIoT network is composed of sensors, actuators, IIoT devices, and IIoT gateways. As more physical objects will be equipped with different types of sensors, the cybersecurity software should be designed to fit the resource-constrained ones in terms of memory and ability of processing mathematical functions. Consequently, the proposed system used the elliptic curve equation and domain parameters that is recommended by SECG/NIST (e.g., Secp192r1). The Secp192r1 is suitable for IIoT network because the key size is 192 bits (24 bytes) and the time taken for the ECDH to establish the shared secret key is 0.576 s [29]. Furthermore, the fastest known algorithm to resolve the ECDLP for size k requires $0.886\times \sqrt{k}$ steps, this means that to achieve a k-bit security strength, at least 2 × k-bit key size of the curve is needed [32]. Hence, the curve Secp192r1 provides 96-bit security strength. The next generation of sensor (e.g., Lotus) is designed with an enhanced capability such as low power ARM7 Cortex M3 with 32-bit processor, 64 kB SRAM, 512 kB, and 64 MB serial flash memory. The 6LowPAN protocol is used to make direct communication between the IIoT device and sensor nodes. The header length of 6LowPAN is 40 bytes and the length of payload is 127 [40]. Thus, the Secp192r1 equation and domain parameters has been used to implement the key management of ESSC over IIoT network. In order to implement and test the performance of ESSC, Mininet-IoT emulation software has been used to emulate the hardware and communication specifications of IIoT [41]. Figure 3 illustrates the emulation mesh topology which consists of eight sensor devices (sensor1 to sensor8), one IIoT gateway (BaseST1), one mobile IIoT device (IoTDev5), and two intruders (Intrudr6 and Intrudr7). All IIoT devices have two network interface cards which can communicate with the IIoT gateway directly using IPv4 and communicate with IIoT devices using 6LowPAN. The exchange of public keys and messages between all legitimates IIoT devices are implemented using socket program code that integrated with ESSC code. Table 3 presents the detail of the experiment’s configuration and performance comparison metrics. 

### 5.1. Simulation Spoofing Attack and Countermeasure

**Figure 4:**
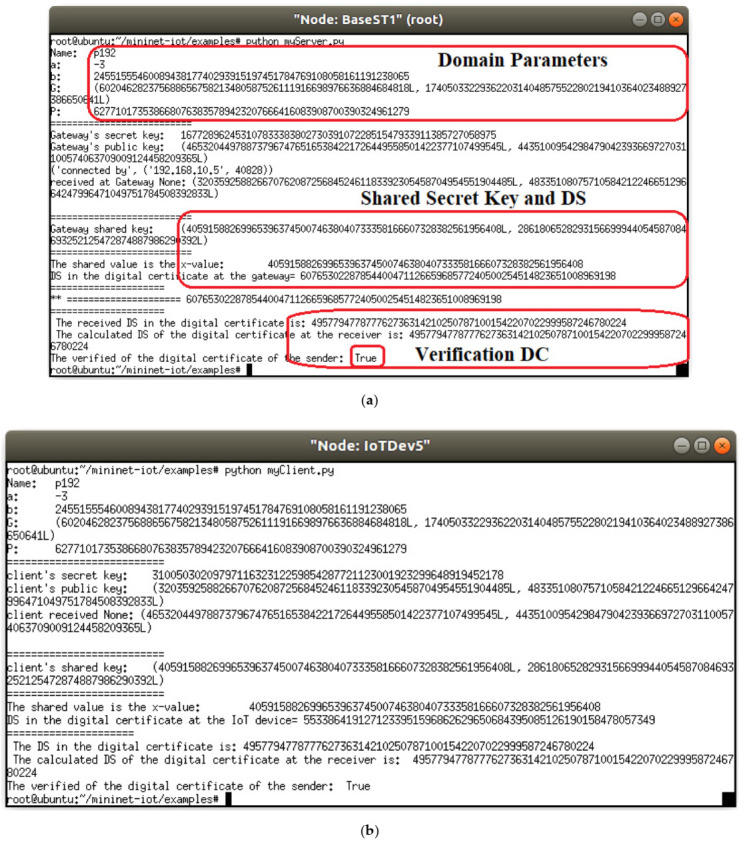
Verification of valid DC (**a**) BaseST1; (**b**) IoTDev5.

**Figure 5:**
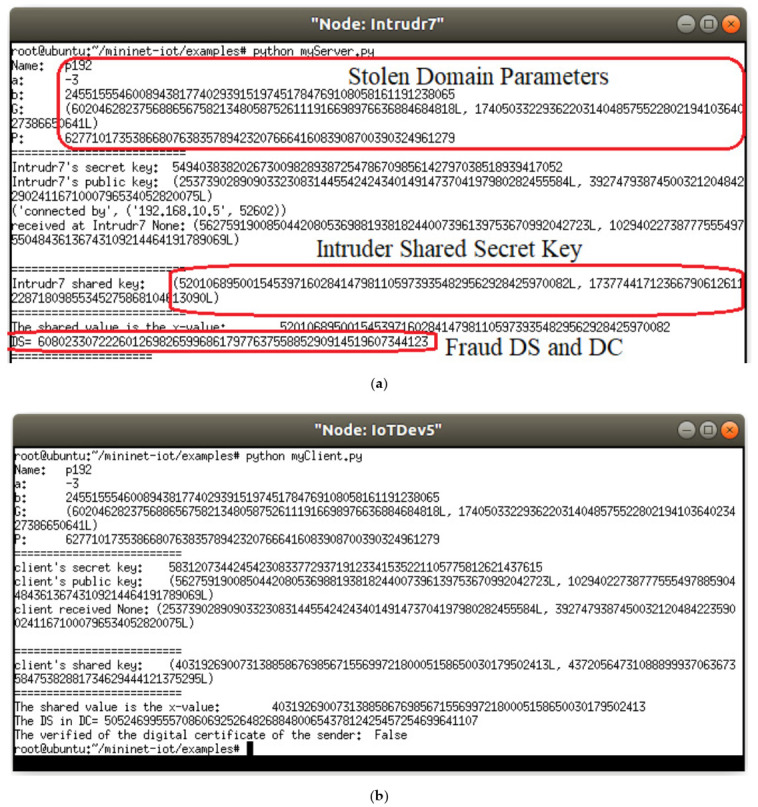
Countermeasures spoofing attack for gateway DC (**a**) Intruder 7; (**b**) IoTDev5.

The IoT device (i.e., IoTDev5) in Figure 3 obtains the DC from the IoT gateway (i.e., BaseST1) which signed the DC using his private key. After that, IoTDev5 verifies the issuer of DC using the stored public key of IoT gateway and ECDH key exchange. Figure 4 shows the verification of genuine CAC signature upon receiving the DC from the IoT gateway. The two parties, IoTDev5 and IoTDev5, authenticates each other in Figure 4. Let us assume the following scenario:

The intruder 7 (i.e., Itrudr7) used a man-in-the-middle and stolen-verifier attacks to obtain the transferring DC between the BaseST1 and IoTDev5. Thus, the intruder 7 can pretend to be the issuer of the DC (i.e., pretend to be the CAC). As can be shown in Figure 5, the ESSC mechanism on IoTDev5 can defend against these two attacks by using the verification of digital signature in the DC. The public key will be used to calculate the shared secret key which is not sent through the channel between the parties of IIoT system. Therefore, the intruders have no chance to spoof this key. Moreover, the shared secret key uses further hash function to remove weak bits in the specific case of domain parameters selection. The hash function for the ephemeral shared secret key creates random number that used to make the session identity code sporadically changes. Even if the digital certificate is stolen, the intruder needs to resolve the digital signature of ***DC*** in order to gain access to the IIoT network. This is basically due to the digital signature between both parties of the session is mandatory to be used in the validation process between IoTDev5 and BaseST1. Hence, IoTDev5 decides the DC sent by Intrudr7 is a fraud DC as can be shown in Figure 5.

### 5.2. Performance Evaluation

The performance evaluation of using of ESSC (digital certificate and cryptographic) has been analyzed in terms of storage, communication and computation costs.

#### 5.2.1. Storage Cost Analysis

The total cost of memory usage (*Tot*) in IIoT device can be estimated as the summed cost of sensed data (*S_data_*), communication (send/received message) data (*C_data_*), and the program source code (*SC*) in time unit *t*. The equation of storage cost can be expressed as:

(17) $$T_{t}=\sum_{t=0}^{n}{\left(S_{data}+C_{data}\right)}_{t}+SC$$

#### 5.2.2. Computation Cost Analysis

The computation cost (*C_P_*) can be calculated as the multiplication of the number of steps per execution (s/e) for all functions (*S_te_*) and the energy consumption of each step (*E_S_*). The equation of computation cost can be expressed as:

(18) $$C_{P}=S_{te}\times E_{S}$$

#### 5.2.3. Communication Cost Analysis

The communication cost (*C_m_*) can be calculated as the total number of packet overhead (*P_H_*) that is required to implement an algorithm (digital certificate or cryptographic) multiplied by the energy consumption of each packet (*E_P_*). The equation of communication cost can be expressed as:

(19) $$C_{m}=P_{H}\times E_{P}$$

### 5.3. Comparative Analysis with the State of Art Solutions

The comparison between the proposed ESSC and certificate-based authentication in TLS/DTLS handshake (CAH), Lightweight X.509 Digital Certificates (LX.509), ECIES_AES, and ECIES_Ra algorithms has been studied. The python source codes of all baseline algorithms are downloaded from [42] website and implemented on the Mininet-IoT emulator. Each scenario of the following experiments is repeated 20 times and each time, 1000 messages are transferred, after which the average results have been calculated for all algorithms. The confidence level of the results is 95%. Furthermore, the *cProfile* and *memory_profiler* Python programs have been used to estimate the deterministic profiling for all algorithms in terms of storage, communication and computation costs.

#### 5.3.1. Performance Comparison between ESSC Digital Certificate and Baseline Algorithms

**Figure 6:**
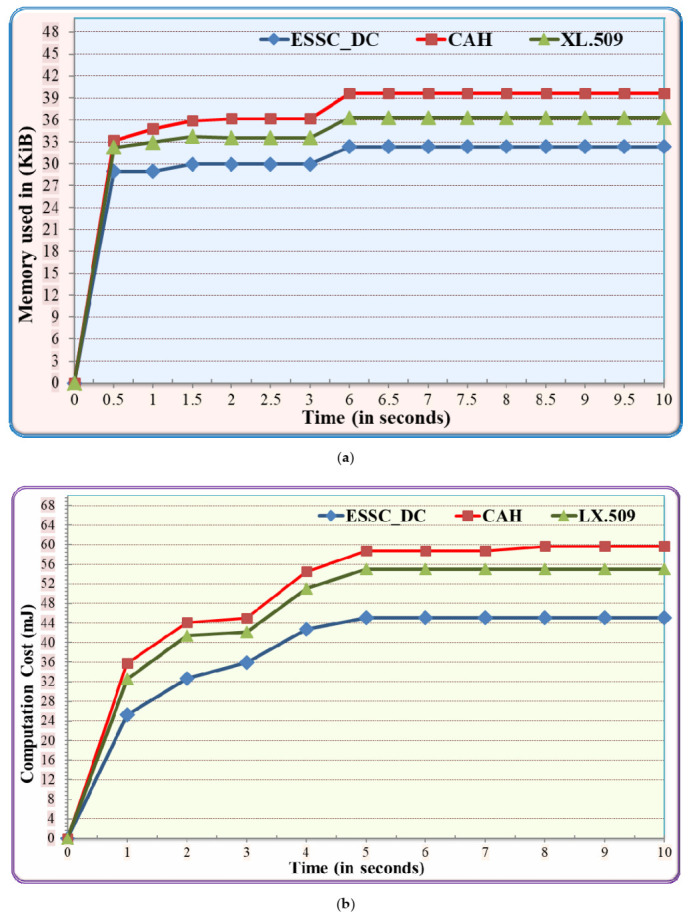

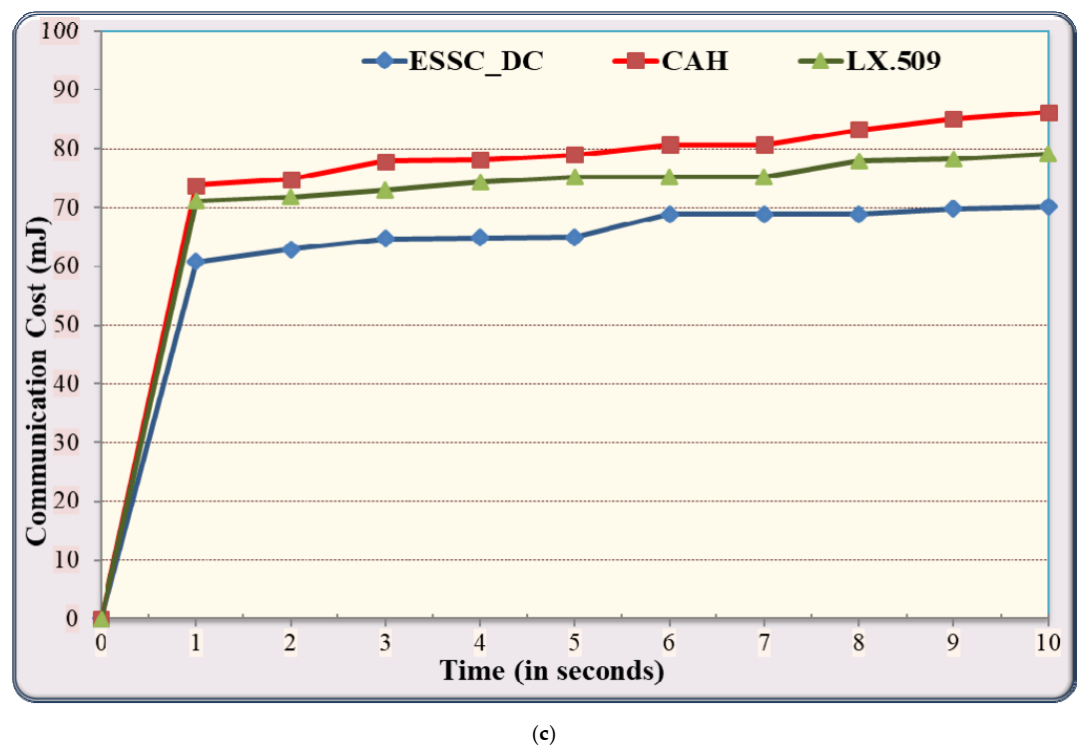
Performance comparison between ESSC digital certificate and baseline algorithms on IIoT (**a**) Execution Time; (**b**) Function Calls; (**c**) Energy Consumption.

The performance of using ESSC digital certificate (ESSC_DC) has been evaluated based on storage, communication and computation costs. Meanwhile, the results have been compared with CAH and L.509 benchmark algorithms. As can be seen in Figure 6, the performance results of the three algorithms have increased sharply at the starting point (0–1 s) due to the initialization phase of the three algorithms. After that, the performance results have been increased gradually with the simulation time due to the certification management between the authority parties. In Figure 6a, the ESSC_DC experiences on average 17.7% less memory usage compared to CAH and it experiences on average 10.9% less memory usage compared to LX.509. Moreover, Figure 6b illustrates that ESSC_DC experiences on average 23.8% and 18.1% less computation cost compared to CAH and LX.509 respectively. Also, Figure 6c shows that ESSC_DC consumes on average 16.9% and 13.1% less communication cost compared to CAH and LX.509 respectively. The previous results are mainly achieved due to the following reasons. Firstly, ESSC_DC spends a smaller number of message communication overhead compared to the LX.509 and CAH. CAH and LX.509 consumes more resources due to the size of digital certificate includes unnecessary fields such as Issuer and subject unique ID, Subject, Extensions. Moreover, CAH and LX.509 used concise binary object representation (CBOR) to encode and ultimately compress the profiled X.509 certificate. Consequently, the compress and decompress functions consume more computation cost and increase the communication overhead.

#### 5.3.2. Performance Comparison between ESSC Cryptographic and Baseline Algorithms

**Figure 7:**
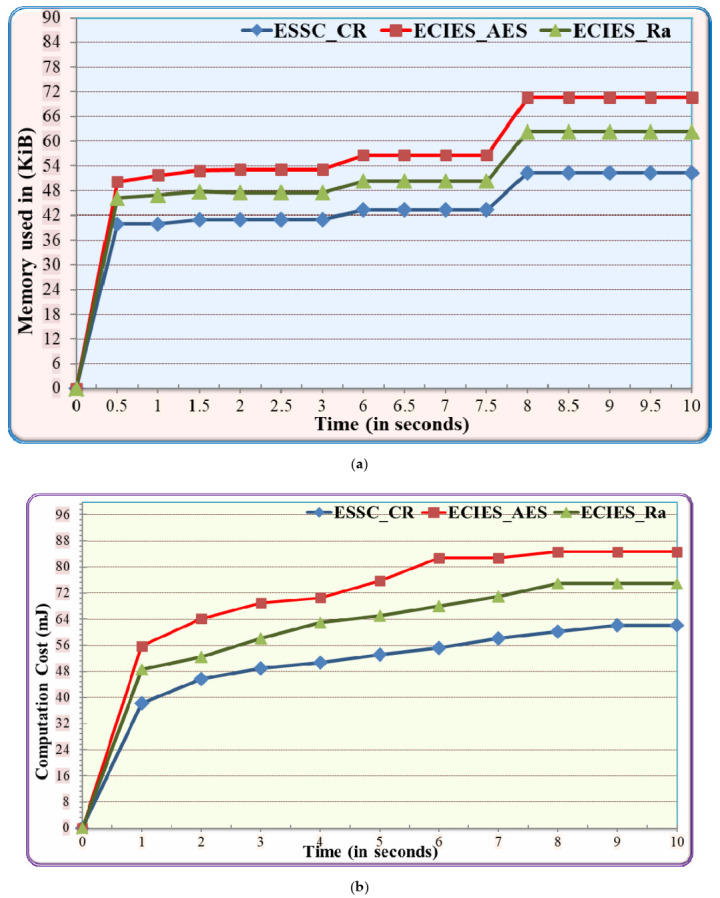

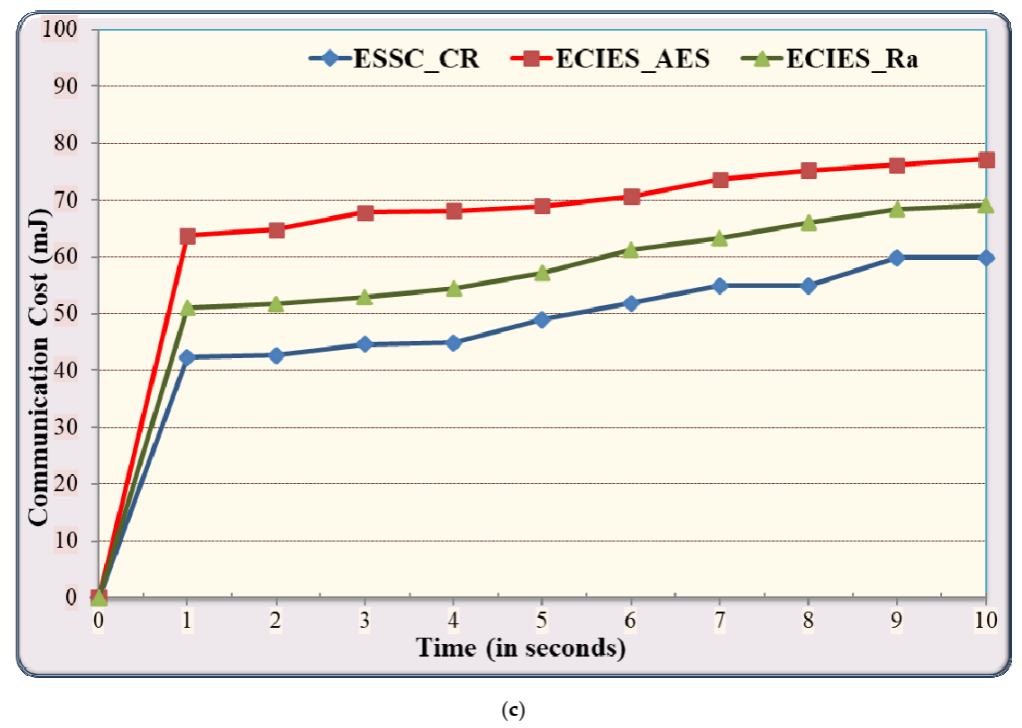
Performance comparison between ESSC cryptographic and baseline algorithms on IIoT (**a**) Execution Time; (**b**) Function Calls; (**c**) Energy Consumption.

In this experiment, the performance of the ESSC cryptographic algorithm (ESSC_CR) has been evaluated based on storage, communication and computation costs. The results have been compared with EDIES_AES, and ECIES_Ra benchmark algorithms. As illustrated in Figure 7, the performance results have been affected by encryption and decryption functions at ESSC_CR, EDIES_AES, and ECIES_Ra. In Figure 7a, the ESSC_CR experiences on average 23.9% and 14.6% less memory usage compared to EDIES_AES and EDIES_Ra respectively. Furthermore, Figure 7b depicts that the ESSC_CR experiences on average 29.2% and 17.95 less computation cast compared to EDIES_AES, and ECIES_Ra respectively. Also, Figure 7c shows that ESSC_CR consumes on average 28.6 and 17.95% less communication cost compared to EDIES_AES and EDIES_Ra respectively. Overall, the results in Figure 7 show that the ESSC_CR outperforms EDIES_AES and ECIES_Ra in terms of storage, communication, and computation costs. This is primarily due to the following reasons. Firstly, ESSC_CR consumes less memory usage to encrypt the message based on its secure and effective hash function idea and modular multiplication. Secondly, the ESSC_CR consumes few computation steps due to the fact that ESSC_CR does not execute several rounds or complex mathematical in the encryption/decryption function which means less function calls and execution steps per each function. Finally, ECIES_Ra uses stream ciphertext which is complex to be implemented correctly in IIoT while EDIES_AES uses several rounds to implement encryption/decryption function. Overall, ESSC_CR and ESSC_DC outperform the baseline security protocol in terms of storage, communication, and computation costs.

## 6. Limitations of the Implementation of ESSC in IIoT

ESSC provides a promising and emerging security solution for IIoT networks, which enables secure, intelligent industrial operations and achieves high trusted productivity without requiring human intervention. Hence, the proposed ESSC encourages business leaders in civilian and military production facilities to choose implementation of IIoT in a smart manufacturing environment. Cyberattacks can cause catastrophic consequences to production processes, damage to instruments and equipment and financial losses. The potential compromised of attacker on ESSC can occur on the IIoT gateway (CAC) which has been assumed to have a robust security mechanism. Although the ESSC was carefully developed for IIoT, there were some of unescapable hardware constraints. Firstly, the ESSC faces a long process when applied to any IIoT device that uses an 8-bit microcontroller due to the limitation of mathematical calculations that are needed in ESSC. This means IIoT network devices with 32-bit or 64-bit microcontroller are preferred to implement ESSC algorithms. In 2019, the IIoT 32-bit microcontroller market has experienced more than 50% (57.1%) growth compared to all other types of microcontroller. This means a gradual transformation from 8-bit MCUs to 32-bit and 64-bit MCUs is occurring; hence, ESSC provides a promising and emerging security solution for IIoT networks. Finally, the scalability and capacity of IIoT networks require more research and study to deal with the hardware specifications of IIoT which are not well-defined yet in the standard reported IIoT networks. 

## 7. Conclusions and Future Work

The proposed ESSC algorithm has been presented, proven in the ROM and compared with standard digital certificate and lightweight cryptographic schemes. In this paper, the vulnerability of the standard digital signature mechanism has been described. ESSC utilizes ECDH to provide secure digital certificate management and symmetric cryptographic schemes for the IIoT and it solves the problem of verification of the true identity of the sender. Also, the threat model has been defined and the cybersecurity analysis shows that the ESSC has been proven secure against CPA, CCA, and RKA. Moreover, the emulation results that obtained in this research demonstrate the efficiency and effectiveness of ESSC performance in terms of storage, communication and computation costs compared with the standard baseline digital certificate and cryptographic mechanisms. The future work of this research will focus on developing an effective key management to replace the ECDH that has been used in ESSC over IIoT networks.

## Figures and Tables

**Table 1:** Summary of related works.

Approaches	Date of Publishing	Methodology and Features	Limitations
Forsby et al. [15]	2017	The authors proposed lightweight X.509 (LX.509) which is compatible with the X.509 standard.	It uses concise binary object representation (CBOR) to encode and ultimately compress the profiled X.509 certificate.
ECDSA [16]	2001	It is the elliptic curve analogue of the digital signature algorithm.	Slowness design flaws and insufficiently defensive.
SCDSA and MPS-SCDSA [17]	2018	It secures communication between smart devices in human centered IoT.	It needs high processing resources and consumes extra energy.
Yasir et al. [18]	2017	It proposes a lightweight security mechanism based on ECC and ElGamal for encryption and decryption over public-key infrastructure (EEoP).	It lacks the security and adversary mode analysis.
Alizai et al. [19]	2018	It proposes a secure multi-factor authentication which uses digital signatures and device capability to authenticate a device on IoT.	The parameters of the digital signature scheme are not adequate to claim authenticity.
KMP [20]	2017	It combines implied certificates with ECDH exchange for verifying authentication and key generation	It consumes more resources due its use of implied certificates.
B. Seok et al. [22]	2020	It proposes a secure D2D communication based on ECC and AEAD ciphers to cover resource-constrained IoT devices.	It lacks security and adversary mode analysis.
SIT [23]	2017	It uses a mixture of feistel and a uniform substitution-permutation network.	It consumes more resources due to using complex permutation.
NTSA [24]	2019	It proposes a dynamic key confusion for each round of encryption for the transfer a text files through the IoT network.	It is limited to text file transmission.
Shah et al. [25]	2017	It proposes a combination of encryption algorithms, Diffie–Hellman, and a multifactor authentication system to share a secret key over the network.	It lacks security and adversary mode analysis.
IBE-ECC [26]	2015	It proposes a lightweight one-time password (OTP) scheme based on Elliptic Curve Cryptography.	It depends on a pre-shared key based Diffie-Hellman exchange which is not sufficient to create secure encryption.
M. Ayoub et al. [28]	2020	It proposes a secure authentication and encryption based on improved ECC that used biometric parameters.	The biometric parameters are vulnerable to unpredicted errors.
Adeel et al. [29]	2019	It merges two algorithms: ECC to select the key pair, and Elgamal to exchange the secret key.	It lacks the adversary mode analysis.

**Table 2:** Frequently used notation.

Notation	Meaning	Notation	Meaning
C	Ciphertext	IIoT	Industrial Internet of Things
CAC	Certificate authority center (i.e., Gateway)	*M*	Plaintext message
CCA	Chosen-cipher attack	*m*	Converting M to the integer number
CPA	Chosen-plaintext attack	*n*	order of G
*d*	Private key	*O*	An extra point at infinity of the curve
*d_C_*	Private key for CAC	*P*	Modular prime
*d_S_*	Private key for source	*PFS*	Perfect forward secrecy
D	Destination node	*Q*	Public key
DA	Direct access	*Q_C_*	Public key for CAC
DC	Digital Certificate	*Q_S/C_*	Public key for Source or CAC
DC_C_	Digital Certificate for CAC	*Q_S_*	Public key for Source
DC_S_	Digital Certificate for source IIoT device	*R*	Secure random number
DS	Digital signature	*RDC*	Request digital certificate
ECC	Elliptic curve cryptography	*S*	Source node
ECDH	Elliptic Curve Diffie Hellman	*SSK*	Shared secret key
ESSC	Effective secure symmetric cryptography	*X*_1_	The X coordinate of random point E
*G*	Base point generator	*X_K_*	Shared secret key
*h*	Subgroup cofactor	${\mathbb{Z}}_{n}$	The set of integer number modulo *n*

**Table 3:** Experiment Configuration.

Parameter	Values
MAC and PHY	802.15.14_hmsim and 802.11_hmsim
Propagation Model	Shadowing
Path loss exponent	3.0
Shadowing deviation (dB)	3.0
Event area	(1000 m × 900 m)
Cover of IoT device	150 m
Cover range of BaseST1	250 m
Traffic Emulator	TCP Socket client/server; 1000 messages
Performance metrics	Computation Cost, Storage, Energy Consumption
ECDH curve	Secp192r1
Key size	192 Bits (24 bytes)
MTU of the message	127 bytes
Emulation duration	1000 s

## Data Availability

Not applicable.
